# Quantitative monitoring and modelling of retrodialysis drug delivery in a brain phantom

**DOI:** 10.1038/s41598-023-28915-3

**Published:** 2023-02-02

**Authors:** Etienne Rognin, Niamh Willis-Fox, Ronan Daly

**Affiliations:** Department of Engineering, Institute for Manufacturing, University of Cambridge, 17 Charles Babbage Road, Cambridge, CB3 0FS UK

**Keywords:** Targeted therapies, Biomedical engineering

## Abstract

A vast number of drug molecules are unable to cross the blood-brain barrier, which results in a loss of therapeutic opportunities when these molecules are administered by intravenous infusion. To circumvent the blood-brain barrier, local drug delivery devices have been developed over the past few decades such as reverse microdialysis. Reverse microdialysis (or retrodialysis) offers many advantages, such as a lack of net volume influx to the intracranial cavity and the ability to sample the tumour’s micro-environment. However, the translation of this technique to efficient drug delivery has not been systematically studied. In this work, we present an experimental platform to evaluate the performance of microdialysis devices in reverse mode in a brain tissue phantom. The mass of model drug delivered is measured by computing absorbance fields from optical images. Concentration maps are reconstructed using a modern and open-source implementation of the inverse Abel transform. To illustrate our method, we assess the capability of a commercial probe in delivering methylene blue to a gel phantom. We find that the delivery rate can be described by classical microdialysis theory, except at low dialysate flow rates where it is impacted by gravity, and high flow rates where significant convection to the gel occurs. We also show that the flow rate has an important impact not only on the overall size of the drug plume, but also on its shape. The numerical tools developed for this study have been made freely available to ensure that the method presented can be used to rapidly and inexpensively optimise probe design and protocol parameters before proceeding to more in-depth studies.

## Introduction

Bioavailability in the brain of systemically-administered drugs is generally poor due to the blood-brain barrier. This has largely hindered the development of new therapeutic options, especially for the treatment of brain cancers. Many targeted drug delivery techniques have been proposed to overcome this challenge^1,2^. For non-resectable tumours in particular, the possibility to insert catheters or implants, analogously to standard biopsy procedures, has been explored in a variety of ways, for example in Convection-Enhanced Delivery (CED)^3–6^, electrokinetic^7^ or electrophoretic^8^ delivery, gel implants^9,10^, or retrodialysis^2,11–18^.

**Figure 1:**
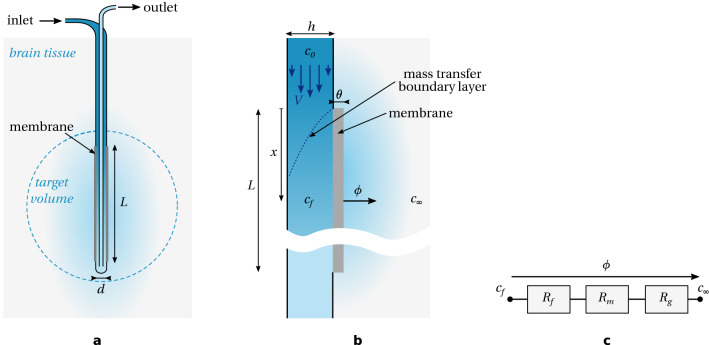
Geometry and theory of operation of retrodialysis. (**a**) Overview of the geometry (cross-section); (**b**) Details, symbols and notation; (**c**) Circuit analogy.

CED, the leading experimental treatment among these techniques, consists of injecting drug solutions with a catheter directly to the tumour site. A major challenge is to reach a reasonable infusion rate while avoiding over-pressure in the intracranial space and reflux along the catheter port^19^. By contrast, when using a microdialysis probe in reverse mode the drug solution (dialysate) flows through a closed channel and leaves at an outlet, as depicted in Fig. 1. Therapeutic agents are delivered by diffusion through a membrane with no net fluid flow and therefore no hydrostatic pressure increase. The absence of reflux along the insertion pathway is also beneficial for wound healing after surgery. An additional advantage of microdialysis is the ability to sample the tumour micro-environment and adapt the treatment strategy^12,17,20^. However microdialysis probes and accessories have been designed and optimised to measure solute concentrations and their translation to efficient drug delivery devices has not been systematically studied. In particular, a system to measure the release of a dye as a drug model to a brain tissue phantom would be beneficial for the rapid optimisation of the design and operating conditions of microdialysis probes in reverse mode.

In the context of CED, Sindhwani et al.^21^ developed an instrument to study the delivery and reflux of a dye solution to an agarose gel. Their methodology relies on carefully controlled absorbance images and a tailored concentration reconstruction algorithm. Since it is entirely based on optical imaging it does not require the use of expensive equipment such as X-ray or MRI scanners. We show further improvements to this technique, such as the use of a band-pass optical filter which extends the applicability of Beer-Lambert law of absorbance and increases the versatility of the system by being able to quickly adapt to different dyes. We also employ a telecentric lens which is beneficial for reconstructing concentration maps of wider areas. Importantly, we use a Python environment and open-source packages for the entire data analysis pipeline. Both the data used to support this study and the analysis scripts are freely available and will support the rapid development of the field.

The paper is organised as follows: first we reexamine the theory of microdialysis mass transfer, and the developments needed from the perspective of drug delivery. Then we present an experimental instrument to measure the release of a dye in a brain phantom and provide a detailed description of the data processing framework. Finally we illustrate and discuss our methodology by performing a series of experiments on a commercially-available probe.

## Theory

Solute transfer from a low Reynolds number capillary flow through porous walls is an archetypal problem in life sciences^22^. It has been discussed in the context of microdialysis by many authors^23–28^, therefore we only report here the main assumptions and scaling laws. However, before doing so, we shall emphasise the points specific to a reverse mode of operation which are not straightforward from previous analyses. First, in probing mode, the quantity of interest is likely a concentration in the vicinity of the probe. In reverse mode the concentration of the drug (bioavailability) is important but we can argue that we are also interested in the total mass delivered to the tissue, and at which rate this is achieved. Second, in probing mode we expect concentration gradients not to extend too far from the membrane. In reverse mode we would like to target a larger volume in order to reach tumour edges. Therefore we will discuss the impact of transient concentration gradients in the gel. Finally, we highlight the role of the flow rate (or equivalently the fluid velocity) in the channel. Not only does it drive the overall delivery rate, but it also affects the shape of the drug plume, as we will see in the “Results and discussion” section.

The geometry of the probe is sketched in Fig. 1a,b. The probe has an active length *L* (length of the membrane in the direction of the flow), and an outer diameter *d*. We consider a probe with a large aspect ratio *L*/*d*. Although we are dealing here with a concentric cylinder design with an annular flow along the membrane, the following scaling analysis also applies to other geometries such as linear capillaries or rectangular channels, so long as the aspect ratio of the probe is large. Other parameters are the channel depth, *h*, the membrane thickness, $\theta$, the average flow velocity, *V*. The drug is supplied at concentration $c_{0}$, the flow-averaged concentration (average convected concentration) in a cross-section of the channel is $c_{f}$, and the drug concentration in the gel far from the probe is $c_{\infty }$. We will assume no initial drug in the gel, therefore $c_{\infty }=0$. The effective diffusivity of the drug is $D_{0}$, $D_{m}$, and $D_{g}$ in the flow, membrane and gel, respectively.

Considering a thin slab at position *x* along axis, the radial mass flux per unit area, $\phi$, at the probe surface can be written as^25^:

1 $$\begin{aligned} \phi (x)=\frac{c_{f}(x)-c_{\infty }}{R_{f}(x,V)+R_{m}+R_{g}(t)} \end{aligned}$$

where $R_{f}$, $R_{m}$, and $R_{g}$ are the resistance to mass transfer due to the flow, the membrane and the gel respectively, see Fig. 1c. Dependence upon *x*, *V* and time *t* have been emphasised in this equation and shall be discussed now.

First, in the flow, it is assumed that the concentration gradient (and therefore mass transfer by diffusion) is mainly radial. This is valid provided that the Péclet number relative to the active length is large:

2 $$\begin{aligned} Pe_{L}=\frac{VL}{D_{0}}\gg 1 \end{aligned}$$

In practice, this sets a minimal flow rate for the flow to have any effect on the delivery mechanism, compared for example to drug diffusion from a gel implant. Regarding the mass transfer from the bulk flow to the membrane inner surface, we can distinguish two regimes of flow: a moderate flow where the radial concentration gradient spans the entire depth of the channel along the majority of the active length. In other words, the mass transfer boundary layer (see Fig. 1b) is as large as the channel depth. This is the case if the Péclet number relative to the channel depth is small or of the order of unity:

3 $$\begin{aligned} Pe_{h}=\frac{Vh}{D_{0}}\lesssim 1 \end{aligned}$$

This is the typical assumption in previous microdialysis analyses^25^. The flow-averaged concentration $c_{f}$ is then a function of *x* because the drug is depleted as it flows past the membrane. Conversely, the mass transfer resistance of the flow is constant and scales as:

4 $$\begin{aligned} R_{f}\sim \frac{h}{D_{0}} \end{aligned}$$

The second regime is a strong flow with $Pe_{h}\gg 1$ where the mass-transfer boundary layer is actually smaller than the depth of the channel and the flow-averaged concentration remains approximately constant. It should be emphasised that this situation is neither unlikely nor contradictory with a low Reynolds number hypothesis. It is not unlikely because large therapeutics such as nanoparticle drug carriers or biomolecules have very small diffusivity coefficients and therefore high Péclet numbers can be achieved even with very low flow rates. It is also compatible with a low Reynolds number hypothesis, because the Schmidt number which is the ratio of the Péclet number to the Reynolds number, but also the ratio of solvent momentum diffusivity to solute mass diffusivity, is always large in liquids, even for small drugs. The boundary layer thickness, $\delta$, can be estimated to grow as^29^:

5 $$\begin{aligned} \frac{\delta }{h}\sim \left( \frac{x}{h}\right) ^{\frac{1}{3}}Pe_{h}^{-\frac{1}{3}} \end{aligned}$$

Therefore:

6 $$\begin{aligned} R_{f}(x,V)\sim \left( \frac{hx}{D_{0}^{2}V}\right) ^{\frac{1}{3}} \end{aligned}$$

The resistance of the membrane, $R_{m}$, is constant and given only by its geometry and materials properties^25^. Finally we consider the resistance of the external gel, $R_{g}$. In this phantom setting characterised by the absence of a sink term for the drug or dye, it is appropriate to assume a transient concentration field in the external medium, at least during a time $t\sim L^{2}/D_{g}$ which is of the order a several days in examples that will be examined below. Therefore the idea of mass transfer resistance must be considered with care. It is clear that at the beginning of the delivery experiment the gel offers no resistance to the diffusion of dye. If a steady-state concentration is eventually reached, and assuming an infinitely large gel, the resistance would be:

7 $$\begin{aligned} R_{g}=\frac{d}{\alpha D_{g}} \end{aligned}$$

where we recall that *d* is the probe outer diameter, and $\alpha$ is a numerical coefficient of the order of unity. If the probe was a spherical object then $\alpha =2$, but for the probe under investigation here $\alpha \sim 0.4$ (from numerical simulations not reported here). This expression gives an upper bound of the resistance. To assess how fast an effective resistance would grow, we can consider the gel as a cylindrical shell of inner diameter *d* and outer diameter $d+2\sqrt{D_{g}t}$, with $\sqrt{D_{g}t}$ being the characteristic length of diffusion after time *t*. Then the resistance of this cylindrical shell scales as:

8 $$\begin{aligned} R_{g}(t)\sim \frac{d}{2D_{g}}\ln \left( 1+\frac{2\sqrt{D_{g}t}}{d}\right) \end{aligned}$$

Consequently at short times ($t\ll d^{2}/D_{g}$), $R_{g}$ would appear to grow as $\sqrt{t}$, but at longer times it has a much slower logarithmic growth. In particular, if seen from a narrow time window this gel resistance would appear almost constant.

The conclusion of this discussion is that, for the moderate flow ($Pe_{h}\lesssim 1$), the drug concentration in the channel $c_{f}(x)$ is found by solving the differential equation:

9 $$\begin{aligned} \frac{\text{d}c_{f}}{\text{d}x}=-\frac{P}{Q}\phi (x)=-\frac{P}{QR}c_{f}(x) \end{aligned}$$

where *P* is the perimeter of the membrane, *Q* the volumetric flow rate in the channel, and $R=R_{f}+R_{m}+R_{g}$ (the time-dependency of $R_{g}$ will be discussed again in the results section). The total delivery rate, $\Phi$, is then:

10 $$\begin{aligned} \Phi =P\int _{0}^{L}\phi (x)\text{d}x=c_{0}Q\left( 1-\exp \left( -\frac{PL}{QR}\right) \right) \end{aligned}$$

At this point it is interesting to consider how the mass flux varies with flow rate. When *Q* is small (but keeping $Pe_{L}\gg 1$), then $\Phi$ is small as well, because although mass transfer has plenty of time to occur over the length of the membrane, the available flux is still $c_{0}Q$. On the other hand, when *Q* is large then $\Phi$ is independent of the flow rate and $\Phi \rightarrow c_{0}PL/R$. Considering the mechanics of the flow more closely, it is clear that this expression is not accurate at high flow rate because the resistance of the fluid is negligible: $R_{f}\rightarrow 0$ and $c_{f}(x)\approx c_{0}$, and therefore the maximum delivery rate is in fact given by^23^:

11 $$\begin{aligned} \Phi _{\max }=\frac{c_{0}PL}{R_{m}+R_{g}} \end{aligned}$$

A more accurate transition to this asymptotic maximum value at strong flows ($Pe_{h}\gg 1$) is found by integrating the mass balance along the variable resistance $R_{f}(x)$ given by Eq. (6). Yet if the mass transfer resistance is still dominated by the membrane and the gel then using a more complex model is of limited value and expression (10) with an adjusted resistance is preferred.

Finally, we can define a drug delivery efficiency, $\eta$, as being the ratio of the mass flux out of the probe to the available flux from the reservoir:

12 $$\begin{aligned} \eta =\frac{\Phi }{c_{0}Q} \end{aligned}$$

We can see $\eta$ as the analog of the *dialysate extraction fraction* defined for concentration probing^25^. In a drug delivery scenario, going to the extreme low or high flow rates is not beneficial as in the former drug is delivered very slowly albeit efficiently, and in the latter almost all of the drug goes to the waste, unless recirculation is conceivable. It is therefore appropriate to use a regime where the mass fluxes out of the probe and to the waste are of the same order of magnitude, targeting $\eta \sim {{1}/{2}}$.

**Figure 2:**
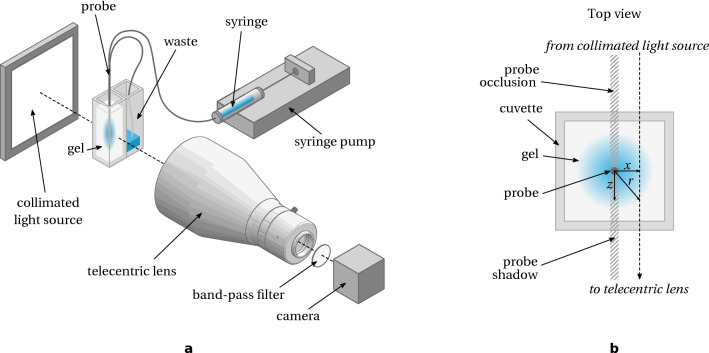
Experimental setup. (**a**) Overview; (**b**) top view of the gel phantom.

## Experimental

### Sample preparation and instrumentation

The experimental setup is sketched in Fig. 2a. The brain phantom is made of agarose gel, which has been widely used particularly for probe insertion and CED studies^21,30–33^. Agarose powder (Sigma), 60 mg, is dissolved in 10 mL Dulbecco’s phosphate-buffered saline (DPBS) by heating to 100+ °C on a hotplate for 10 min. Once the polymer has dissolved (the solution turns from cloudy to clear), the hot solution is poured into a transparent plastic cuvette (10 mm optical path) and allowed to cool and gel for at least 30 min.

After the gel is set and at room temperature, a hole is made using a pointy metallic rod (outer diameter 0.65 mm). The rod is removed and the micro-dialysis probe (70 MD Bolt Catheter, Mdialysis) is inserted. The probe is flushed with 0.5 mL of DPBS at 15 μL/min. This priming step removes bubbles and impurities from previous storage. Then frame capture is started and 1 mL of a methylene blue solution at a concentration of 0.2 mg/mL in DPBS is infused, first at 15 μL/min during 5 min, then at a fixed flow rate depending on the experiment. The first 5 min at high flow rate ensures that each experiment starts with the entire device filled with the same concentrated solution. It is also the manufacturer’s recommanded procedure. After the experiment, the probe is flushed with 0.5 mL of DPBS at 15 μL/min and stored in buffer until reused for another run. All experiments are done at 20 °C.

A collimated light source (CX0202-WHIIC, Edmund Optics) shines parallel beams through the brain phantom. A telecentric objective lens (TitanTL 0.184X, Edmund Optics) is mounted on a digital camera (Basler Ace acA1920-150um, 2/3” 10-bit monochromatic sensor). The use of telecentric objective eliminates geometric distortion and therefore allows a better 3D reconstruction from the image. A pass-band filter (FB500-40, Thorlabs) centred around 500 nm, with a bandwidth of 40 nm is mounted at the back of the lens. The aim of this filter is to extend the linearity of Beer-Lambert law when absorbance is computed from pixel intensity values, as it will be discussed below. It also gives better versatility of the system by being able to quickly adapt to different dyes depending on their respective absorption characteristics.

Images are recorded at 1 frame per minute with a fixed exposure time for each series (in the range 9 ms to 11 ms). Images are processed using a Python environment and open-source packages. The next subsection describes how concentration or mass information can be extracted from images.

### Data analysis

At a specified wavelength, $\lambda$, and neglecting internal reflection and scattering of light beams, the intensity of the light collected by the sensor, $\psi _{1}$, is related to the intensity of the light source, $\psi _{s}$, by:

13 $$\begin{aligned} \psi _{1}(\lambda )=\frac{\psi _{s}(\lambda )}{10^{A(\lambda )+B(\lambda )}} \end{aligned}$$

where *A* is the absorbance due to the dye and *B* is the absorbance due to the blank gel and the cuvette walls. To get rid of the unknown *B* and $\psi _{s}$, we use a reference setting with no dye (in general the first image of a time series) so that the spectral intensity collected by the sensor is:

14 $$\begin{aligned} \psi _{0}(\lambda )=\frac{\psi _{s}(\lambda )}{10^{B(\lambda )}} \end{aligned}$$

and therefore, the absorbance due to the dye can be found with:

15 $$\begin{aligned} A(\lambda )=-\log _{10}\frac{\psi _{1}(\lambda )}{\psi _{0}(\lambda )} \end{aligned}$$

An important feature of our setup (absent in previous studies^21^) is the use of a band-pass filter which enables the identification of spectral light intensity ratio with pixel value ratios, assuming sensor linearity. Another option could be the use of a monochromatic light source. Under such conditions, we can measure absorbance at time *t* for each pixel referenced by position (*x*, *y*) in the image:

16 $$\begin{aligned} A(t,x,y)=-\log _{10}\frac{I(t,x,y)}{I(0,x,y)} \end{aligned}$$

where *I*(*t*, *x*, *y*) and *I*(0, *x*, *y*) are the pixel intensities at time *t* and at $t=0$ (first image of the stack), respectively. On the contrary, if a band-pass filter is not used and the spectral absorbance of the dye is strongly peaked, then the absorbance is no longer found by Eq. (16).

#### Uniform fields

For uniform concentration fields, such as in a calibration setting, one can use the Beer-Lambert law to relate absorbance to mass concentration, *c*:

17 $$\begin{aligned} A(\lambda )=\frac{\varepsilon (\lambda )\ell }{M}c \end{aligned}$$

where $\varepsilon$ is the molar extinction coefficient, $\ell$ is the optical path length, and *M* is the molar mass.

**Figure 3:**
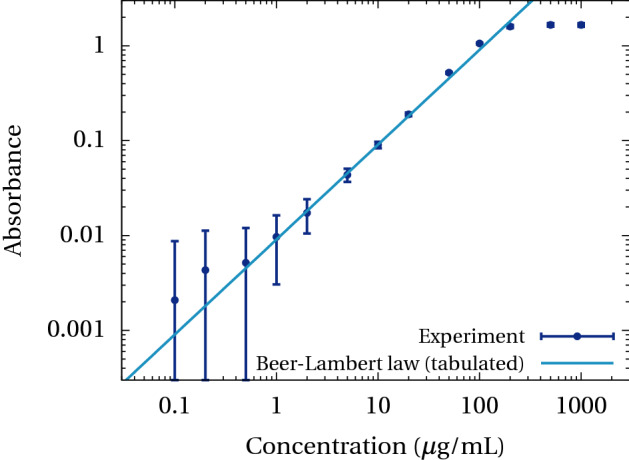
Calibration of methylene blue absorbance around 500 nm with an optical path of 1 cm. Data points represent averages over one cuvette image (per concentration value), error bars show one standard deviation.

A calibration curve for uniform concentrations of methylene blue is shown in Fig. 3. The absorbance of methylene blue in DPBS solutions is measured at a known concentration ranging from 0.1 μg/mL to 1 mg/mL. A tabulated value for the molar extinction coefficient $\varepsilon =2900\,\text{cm}^{-1}\,\mathrm{L/mol}$ produces a good fit of the Beer-Lambert law for absorbance in the range 0.02 to 0.2. Between 0.2 and 1, the absorbance is not strictly linear but the deviation is still small. There is a clear saturation effect above 1. For absorbance below 0.02 the uncertainty is too large for the measure to be conclusive.

#### Non-uniform fields

For non-uniform concentration field, Beer-Lambert law can be expressed in differential form along the optical path parametrised by *z* (see Fig. 2b):

18 $$\begin{aligned} \frac{\text{d}\psi }{\psi }=-\frac{\varepsilon (\lambda )\ln 10}{M}c(z)\text{d}z \end{aligned}$$

To simplify notations, wavelength dependence of $\varepsilon$ will now be implicit. Integrating along the beam and normalising by the reference image gives

19 $$\begin{aligned} \mu (x,y)=\int _{-\infty }^{+\infty }c(z)\text{d}z=\frac{M}{\varepsilon }A(x,y) \end{aligned}$$

with $\mu$ the mass per unit area seen in the image. Therefore, the total mass delivered by the microdialysis probe can be measured by integrating Eq. (19) over the area of interest, regardless of any symmetry property.

Let us now assume that the concentration field has a symmetry axis, here the *y*-axis, and we consider a cross-section at a fixed value of $y=y_{0}$ (see Fig. 2b). We further assume that the concentration field is null beyond a certain radius from the axis (to avoid convergence issues), and that the light beam is in a plane $y=y_{0}$. Integrating again Eq. (18) along the optical path, but also using the change of variable $r=\sqrt{x^{2}+z^{2}}$, with *x* the distance to the symmetry axis, we have:

20 $$\begin{aligned} \frac{M}{\varepsilon }A(x)=2\int _{x}^{+\infty }c(r)\frac{r\text{d}r}{\sqrt{r^{2}-x^{2}}}={\mathscr {A}}(c)(x) \end{aligned}$$

where ${\mathscr {A}}(c)(x)$ is the Abel transform of the concentration field *c*(*r*). Formally, the concentration field can be found from measuring the absorbance and applying the inverse Abel transform, ${\mathscr {A}}^{-1}$:

21 $$\begin{aligned} c(r)={\mathscr {A}}^{-1}\left( \frac{M}{\varepsilon }A(x)\right) \end{aligned}$$

Sindhwani et al.^21^ proposed their own reconstruction algorithm without explicitly referring to the Abel transform. However we see from the above analysis that we can benefit from recent advances in the broader field of computational Abel transform and use robust and validated algorithms. Here we employ the BASEX algorithm^34^ provided with the open-source PyAbel package^35^.

#### Image processing workflow and noise reduction

**Figure 4:**
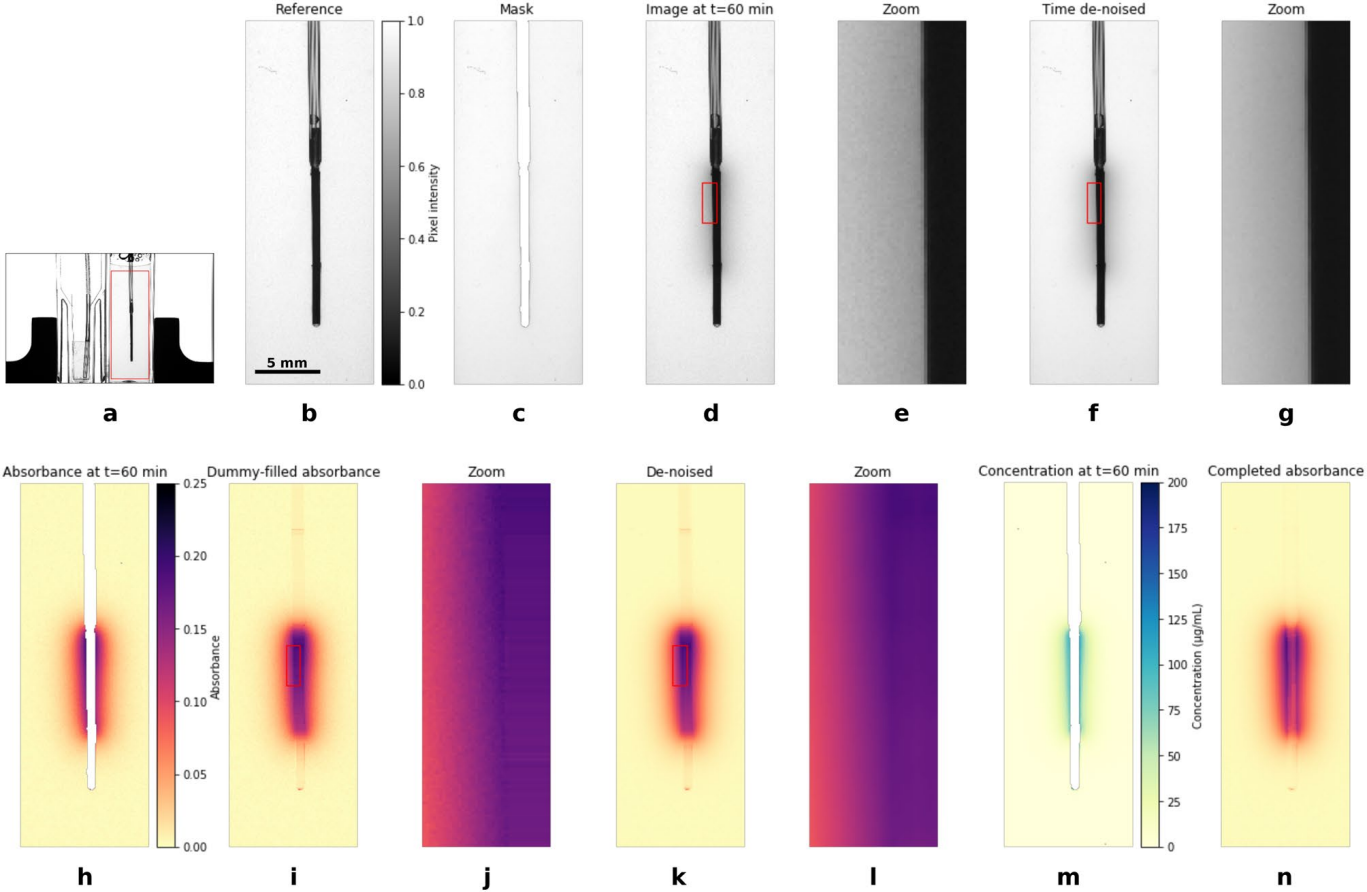
Illustration of the image processing workflow; (**a**) Full view; (**b**) Selected region; (**c**) Mask; (**d**) and (**e**) Image at time $t=60$ min; (**f**) and (**g**) Image at time $t=60$ min after time denoising; (**h**) Absorbance; (**i**) and (**j**) Dummy filled absorbance; (**k**) and (**l**) Absorbance after spatial denoising; (**m**) Reconstructed concentration field; (**n**) Completed absorbance.

The data processing workflow is illustrated in Fig. 4. First, the part of the image corresponding to the brain phantom is selected (Fig. 4a,b). This cropping is applied to each image of the stack. A masking layer (Fig. 4c) is created to locate the probe shadow. This is done in two steps: first, pixels from the reference image with values less than 0.3 are selected, and interior pixels of this selection are also assigned to the shadow. In this way the semi-transparent tubing is correctly masked. Second, any pixel with value less than 0.7 is assigned to the probe shadow, which can also mask opaque dust particles.

We found that noise filtering is not necessary if one is only interested in measuring the mass captured in an area of the image that has many pixels and is away from the probe shadow. However, noise reduction is highly beneficial prior to applying the inverse Abel transform. Images suffer from two main sources of noise: electronic noise of the sensor and light scattering in the gel. The sensor noise is responsible for non-linearity of the dark values (impossibility to have pure black images even with a shutter on) and uncorrelated variability over time in a series of frames capturing the exact same scene. The former is reduced by using short aperture time (here in the order of 10 ms). To reduce time fluctuation, we consider each pixel independently as a time series and apply a wavelet filtering using the *PyWavelets* Python module. The result can been seen in Fig. 4d–g.

Absorbance images (Fig. 4h) are then computed using Eq. (16), where pixels belonging to the probe shadow are ignored. Absorbance images still contain a moderate level of noise. We conclude that the experimental setup is not ideal with respect to pixel-wise absorbance, due to scattering in the gel in particular. To help with further de-noising, dummy absorbance values inside the probe shadow are assigned (Fig. 4i,j). Each absorbance image is then filtered using a 2d wavelet de-noising (from the *scikit-image* Python module) (Fig. 4k,l). The most likely symmetry axis is found by a line-by-line auto-correlation and images are rotated accordingly (step not shown here). Then the inverse Abel transform is applied to compute the concentration field (Fig. 4m). We use the BASEX algorithm^34^ from the PyAbel^35^ module.

Finally, the absorbance inside the probe shadow is computed using the forward Abel transform of the concentration field. This produces an absorbance image (Fig. 4n) we would capture if there was a void in place of the probe. In this way we can estimate the mass in the entire image using Eq. (19).

**Figure 5:**
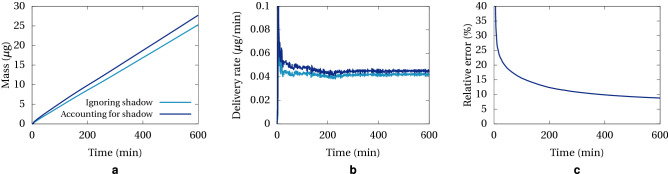
Delivery of methylene blue for a probe flow rate of 0.3 μL/min; (**a**) Mass measured in the image; (**b**) Delivery rate (time derivative of A); (**c**) Relative error when not accounting for probe shadow.

The importance of properly accounting for the probe shadow is illustrated in Fig. 5. Here we report the mass of methylene blue delivered to the brain phantom when the flow rate in the probe is 0.3 μL/min. Ignoring probe shadow simply consists of summing absorbance pixels only outside the shape defined by the mask. We can see in Fig. 5c that the relative error is large at the beginning, when most of the mass is in the immediate vicinity of the probe and therefore a large proportion is occluded. At longer time, the mass is more spread out and the error is less significant. Nevertheless it should be noted that this correction method is accurate under the assumption of axial symmetry. At long time this might not be the case if the cuvette containing the phantom is square and the dye diffuses up to the edges.

In conclusion, we have described a method to monitor the delivery of a drug (or model drug) to a gel phantom. Images captured by a carefully designed optical system are processed to compute a time series of absorbance fields. The mass of drug visible in each image can be derived, and concentration maps are reconstructed using a modern algorithmic implementation of the inverse Abel transform, also providing an estimate of the mass occluded by the probe.

## Results and discussion

### Delivery of methylene blue

**Figure 6:**
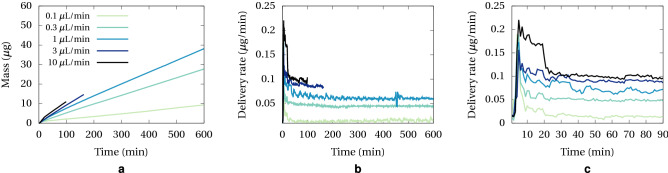
Delivery of methylene blue at various flow rates; (**a**) Mass measured in the image, accounting for probe shadow; (**b**) Delivery rate; (**c**) Detailed delivery rate in the 90 min.

We employed the method described above to assess the performance of a microdialysis probe in delivering methylene blue to a brain tissue phantom. The probe has an axial symmetry design, and a membrane length of 1 cm and external diameter of 0.6 mm. Results are reported in Fig. 6. Figure 6a shows the mass delivered as a function of time, for a range of flow rates in the probe channel. Higher flow rate leads to faster release, as expected from theory (see Eq. (10)) and the released mass appears to grow linearly with time, with no saturation effect observed.

The delivery rate computed by finite difference is plotted in Fig. 6b. The moderate amount of noise can be attributed to residual noise from data filtering and syringe pump variability. Nevertheless, these plots confirm a steady delivery overall at long time. They also feature a peak during the first 20 min, which is shown in more details Fig. 6c. The peak is likely caused by transient diffusion at the very beginning of each experiment when the solution of methylene blue is flushed at high flow rate (15 μL/min) during the first 5 min. In addition, this peak coincides with the initial variation of the mass transfer resistance of the gel, as explained in the theoretical section. After the initial transient, any slow increase of the gel resistance is within the noise, consequently in the remaining we will consider this resistance as a constant.

**Figure 7:**
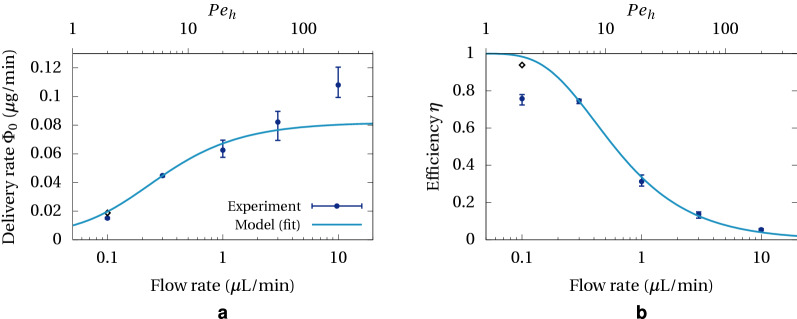
Delivery performance as function of flow rate and comparison with fitted model (Eq. 10). Points ($\bullet$) show average over triplicates, error bars show min and max values. The diamond ($\diamond$) shows an experiment with upside-down cuvette and probe. (**a**) Stead-state delivery rate; (**b**) Delivery efficiency.

The average steady-state delivery rate (i.e. after the initial peak) is measured in each experimental curve, and the delivery efficiency (Eq. 12) is derived. Results are reported in Fig. 7. The model (Eq. 10) is fitted with the experimental data points giving an overall mass transfer resistance $R=0.46\pm 0.06\,\hbox {cm}^{2}\,\text{min}/{\upmu }\,\text{L}$. Two noticeable deviations occur: one at 10 μL/min which is best seen in the delivery rate plot, the other at 0.1 μL/min which is significant in the efficiency plot.

To understand the deviation at 10 μL/min it necessary first to estimate the Péclet number with respect to the channel depth, $Pe_{h}$, for the range of flow rates under investigation. Diffusivity of methylene blue in buffer is of the order of $7\times 10^{-6}\, \hbox {cm}^{2}/\text{s}$^36^. The cross-section area and channel depth, assessed using X-ray tomography, are $0.12 \,\hbox {mm}^{2}$ and 90 μm, respectively. The channel depth is only indicative since images revealed that the outlet tubing is not centred inside the membrane cylinder, leading to a variable channel depth. This eventually leads to having $Pe_{h}\sim 20\times Q$ (*Q* in μL/min). Therefore the Péclet number in the experiments is ranging from 2 to 200, and we can expect a reduced if not negligible contribution of the flow resistance to the overall mass transfer resistance at 10 μL/min. To assess the reduction in resistance, we can calculate the order of magnitude of the low Péclet channel flow resistance (Eq. 4), which is here $\sim 0.02\,\hbox {cm}^{2}\,\text{min}/{\upmu }\,\text{L}$, being 4% of the fitted resistance in Fig. 7a. This effect on its own is unlikely to explain the 30% underestimation in the model at 10 μL/min.

Another suspected cause of increased delivery rate is by net fluid convection to the gel. Cross-membrane flow is caused by a static pressure difference across the membrane. The fluid in the channel has two pathways to return to atmospheric pressure: through the membrane and gel, or through the outlet tubing. Both can be seen as hydraulic resistors in parallel, the resistance of the outlet tubing being in general much lower. Nevertheless, within the timeframe of the experiment, we can see a small rise in the phantom surface of a few pixels. Yet a rise of 4 pixels during one hour corresponds to a fluid flow of 0.18 μL/min and a dye mass transfer rate of 0.036 μg/min, assuming fresh solution at concentration 200 μg/mL is convected. This value is consistent with the gap seen between the model and experimental data.

The occurrence of net convection to the phantom is unexpected with microdialysis probes but can be monitored with this experimental setup. This effect could also be included in the model, as for low Reynolds number flows, the flow rate through a hydraulic resistor is proportional to the pressure difference, and therefore the portion of fluid going through the membrane is always the same. Here this portion is around 2%, so that the added rate of mass transfer would be of the order of $0.02c_{0}Q$. However, we note that at $Q=1$ μL/min, this rate is 4 ng/min, which is within experimental error bars, and therefore modelling this term in the whole range of flow rates is of limited value.

More puzzling is the discrepancy at 0.1 μL/min. Unexplained lower-than-expected performance at low flow rates has been reported in other microdialysis studies^37^. Numerical simulations (not reported here) have ruled out geometry effects such as axial concentration gradient in the gel or eccentric cylinders positions. On the other hand, occasionally we were able to observe a strong stratification of methylene blue in the waste cuvette, with concentrated solution sinking down the bottom during the initial flushing step. To check the influence of gravity, we carried out an additional experiment at 0.1 μL/min but having the cuvette and the probe positioned upside down. The result, reported in Fig. 7 as a black diamond in plots (a) and (b), indicates a much better agreement with the model. The difference can be explained as follows: at low flow rate, the vertical concentration gradient in the channel is maximal because methylene blue is depleted. When the probe is upright (normal position), concentration is higher at the top and therefore the flow is unstable with respect to buoyancy. The imposed velocity may not be large enough to completely overcome this force and part of the inlet fluid sinks faster along the inner wall. When the probe is upside down, higher concentration is at the bottom and there is no reason for buoyancy to influence the flow. This observation should reinforce caution when using microdialysis at low flow rates.

### Concentration maps

**Figure 8:**
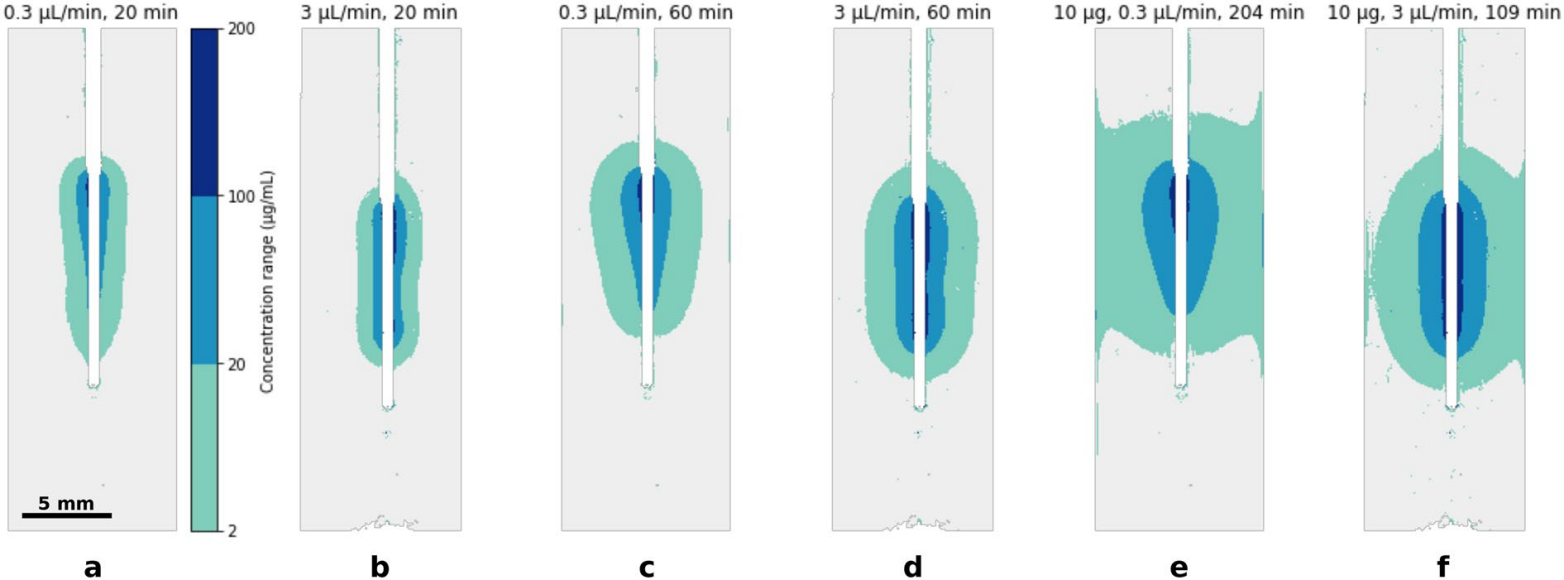
Concentration maps for flow rates of 0.3 μL/min and 3 μL/min; (**a**) and (**b**) After 20 min; (**c**) and (**d**) After 60 min; (**e**) and (**f**) For the same delivered mass of 10 μg.

A major advantage of the method presented here compared to a standard microdialysis setting is the ability to investigate concentration profiles in the brain phantom. Examples of concentration maps are shown in Fig. 8. Although concentration fields obtained at the end of the data processing pipeline have continuous values (see Fig. 4m), a discrete range of colours is used here to ease the comparison of plume size and shape. The thresholds correspond to concentrations larger than 2, 20, and 100 μg/mL. Since the solution concentration is 200 μg/mL, this outlines concentrations in excess of 1%, 10%, and 50% of the model drug solution, respectively. Two flow rates are selected for the comparison: 0.3 μL/min and 3 μL/min, and maps are given for various time steps. Figure 8a,b show concentration at 20 min after the start of the experiment while Fig. 8c,d show concentration at 60 min. Figure 8e,f show different time steps but 10 μg of methylene blue is measured in both images. This would correspond to a strategy where the total mass delivered is the main target.

Starting with the concentration profiles after 20 min (Fig. 8a,b), the plume appears elongated along the probe for both flow rates, and the 1% plume is of similar size. The main difference is that the 10% plume is near the top of the membrane in the 0.3 μL/min case, which is the location where fresh solution flowing from the inlet encounters the membrane. After 60 min, the plume is larger for both flow rates but has a different shape: at 0.3 μL/min (Fig. 8c) the plume has the shape of an upside down pear and methylene blue is more concentrated at the top of the membrane. On the other hand, at 3 μL/min (Fig. 8d), both the 1% and 10% plumes are elongated along the whole membrane length. We also start to see a thin 50% area in the direct vicinity of the membrane.

Finally, we compare the maps at the instant when 10 μg have been released to the gel (Fig. 8e,f). At 0.3 μL/min, the 1% plume is mainly spherical and methylene blue is still more concentrated at the top of the membrane. The 1% plume has extended to the edge of the cuvette. We see in these two images that the reconstruction algorithm has produced a computation artefact where concentration appears to crawl up and down the edges. Yet, the absence of diffusion through to cuvette walls should force the concentration contours to be perpendicular to the boundaries. This artefact is due to the lack of perfect axial symmetry of the real concentration field at long times caused by the cubic shape of the phantom. A larger container could be used to avoid this issue. At 3 μL/min, the plume albeit larger has essentially the same characteristics as at time 60 min (Fig. 8d).

By recalling from Fig. 7b that a flow rate of 0.3 μL/min produces an efficiency of 80%, this means that the channel flow is largely depleted from methylene blue before reaching the end of the active length. Therefore it is expected to see a larger concentration in the gel near the top of the membrane. By contrast, flowing at 3 μL/min is less efficient but keeps a relatively uniform concentration in the channel, producing more elongated concentration profiles. We see from these examples the benefit of being able to monitor the shape and size of the drug plume as it can influence decisions on treatment protocol and strategy.

### Limitations of the present approach

Here we have presented a workflow which benefits from the axial symmetry of the probe in two ways: first, to accurately measure the delivered mass by accounting for the probe shadow, and second, to reconstruct concentration fields from absorbance using the inverse Abel transform. Although the theoretical analysis holds regardless of the detailed design of the probe, measuring the delivered mass accurately or reconstructing the concentration fields can become problematic with the lack of axial symmetry. If the probe is flat, neglecting the shadow might not produce a significant error, however reconstructing concentration would need additional means.

One approach would be to follow a standard computational tomography (CT) method with capturing multiple angles of the scene. The availability of open-source CT libraries makes this approach workable. Nevertheless this would require significant hardware modifications, with a rotating stage and synchronised capture. The delivery process would also need to be slow enough so that variation of concentration field would be negligible during the full rotation time of the cuvette. It may be more beneficial although expensive to use a commercial X-ray CT scanner with a contrast agent matching the properties of the drug under investigation. This would have the additional advantage of exposing the inside of the probe and potential defects such as bubbles which are not visible with our approach.

Another option to deal with non axially-symmetric designs could be to rely on 3D diffusion simulations and solve the inverse problem of finding concentration by matching absorbance fields.

Finally, one can argue that a microdialysis probe can perform rather differently in a real brain than in a phantom. This is true especially if the mass transfer resistance of the brain is much larger than the resistance of the membrane. We can expect that to happen for large vehicles such as nanoparticles. Although we can match diffusion properties of the brain phantom with that of real brains and tumours, it would be still difficult to account for other transport processes such as drug intake or degradation, and interstitial fluid flow. Also, absorbance images rely on the fact that the phantom is transparent, or produces very limited scattering, a property which could be difficult to obtain with more elaborate tissue phantoms. Nevertheless, the method presented here can be used as a part of a quick design optimisation loop before proceeding to more expensive and elaborate in vitro and in vivo experiments.

## Conclusion

In this study we presented a method to measure the retrodialysis delivery of a dye to a brain phantom. Optical images are used to compute absorbance fields and a modern implementation of the inverse Abel transform is employed to compute concentration maps and missing absorbance pixels due to microdialysis probe shadow. The method was used to measure the delivery of methylene blue to an agarose gel with the dye solution flowing through the probe in a range of flow rates. The resulting delivery rate and efficiency can be interpreted using a simple model from the microdialysis literature. Deviations from the model occur for both the lowest and the highest flow rates investigated here. The former is explained by an effect of buoyancy while the latter is due to a net fluid convection to the gel. Reconstructed concentration fields were also compared in different scenarios, showing that the flow rate in the probe affects not only the overall size of the drug plume, but also its shape. The method presented here can be used for quick and inexpensive probe design and protocol iterations before proceeding to more in-depth in vitro and in vivo experiments.

## Data Availability

The datasets generated and analysed during the current study are available in the University of Cambridge Apollo repository, at https://doi.org/10.17863/CAM.88917.
